# Unraveling the Mysteries of Restless Leg Syndrome

**DOI:** 10.7759/cureus.10951

**Published:** 2020-10-14

**Authors:** Mohammad D Memon, Sadaf Faiz, Mitul P Zaveri, Jamal C Perry, Tayná M Schuetz, Ivan Cancarevic

**Affiliations:** 1 Medicine, California Institute of Behavioral Neurosciences & Psychology, Fairfield, USA; 2 Research, California Institute of Behavioral Neurosciences & Psychology, Fairfield, USA; 3 Internal Medicine, California Institute of Behavioral Neurosciences & Psychology, Fairfield, USA

**Keywords:** restless leg syndrome, iron-deficiency, end-stage renal disease, therapy, treatment, dopamine, genes

## Abstract

Restless leg syndrome (RLS) or Willis-Ekbom disease (WED) is an under-diagnosed, chronic, and progressive primary sensory-motor disorder. It is characterized by an uncontrollable urge to move the legs due to uncomfortable and sometimes painful sensations, with a diurnal variation. RLS can lead to severe sleep disturbances, a usual cause of consultation. The pathophysiology is known partially, and it is believed that there is an association between the different variants of genetic mutations combined with dopaminergic and brain iron dysregulation, which plays an important role. The data used for this study were extracted from the articles found in the PubMed database that discuss different gene variants, pathophysiology, and various methods of treatment. They also highlight the role of iron in the pathogenesis of RLS as it is required for the synthesis of tyrosine hydroxylase, which is the rate-limiting step for dopamine synthesis. This review article provides a clinically useful overview of RLS in terms of pathophysiological findings, its genetic associations, and therapeutic options by using the currently available literature. Because RLS presents with vague symptoms and shares similarities with many other diseases, it might be overlooked by many physicians resulting in underdiagnosis and under-treatment. While these discoveries provide a breakthrough in understanding the details of RLS, further studies are recommended as these studies are limited to animal models and provide a limited representation of the general population.

## Introduction and background

"A good laugh and a long sleep are the two best cures for anything," an Irish Proverb.

Sleep is an essential part of our life. It is the time when our body repairs tissue damage and works on the brain to consolidate memories. A study conducted on the American population revealed that about 70% of the adult population is unable to get sufficient sleep once a month, with about 11% reporting insufficient sleep every night [1]. 

Approximately 15% of the population worldwide suffers from restless leg syndrome (RLS) [2]. RLS is a common neurological disorder and is categorized as a sleep disorder. It is characterized by an irresistible urge to move the legs, especially in bed during night sleep, commonly accompanied by unpleasant sensations that subside with movement [3]. The unpleasant, non-painful sensation is generated deep down in the sensorimotor junction during rest. The disease is slowly progressive, and its prevalence increases with age [4]. New evidence suggests that females are twice as likely to be affected as compared to men [5]. Primary RLS (i.e., idiopathic) appears at an earlier age but is not diagnosed until the third decade. It is characterized by a dysfunction of the dopaminergic system [6]. Secondary RLS is associated with some environmental factors and other diseases, such as iron deficiency, end-stage renal disease, pregnancy, diabetes mellitus, and even some neurological diseases [7,8]. RLS is usually diagnosed clinically according to the four main criteria, which include: an urge to move the legs, often associated with unpleasant leg sensations; induction or exacerbation of symptoms by rest; symptom relief on activity; and daily fluctuations in symptoms with worsening in the evening and at night. It can also be diagnosed on polysomnographic recordings as a periodic limb movement with fragmentations of sleep [9]. 

Although the symptoms of RLS were already described as early as the third century BC, it was first classified as a neuromuscular disease by a Swedish neurologist Ekbom in 1945 [10]. Despite recent progress, many individuals remain undiagnosed and untreated for a significant part of their life, as many healthcare providers doubt its validity as an illness. While most of the papers describe RLS as a secondary disease or symptoms of other diseases, a breakthrough came in 2007 when a team of scientists discovered RLS's genetic basis through genome-wide association studies (GWAS) and discovered three polymorphic genes [2]. MEIS1, BTBD9, and MAP2K5/SKOR1 were directly associated with RLS and found in the genome's non-coding part [11]. With further research, secondary variants were also found, which included the alcohol-dehydrogenase 1B (ADH1B) gene, the vitamin D3 receptor (VDR) gene, and the nitric oxide synthase (NOS1) genes [12,13].

The management of RLS has always been challenging due to its multiple etiologies and its wide variability in response to treatment. While dopamine agonists are the first-line agents, many patients will experience worsening of symptoms known as augmentation-paradoxical worsening [9]. In this article, we will be searching the available literature using the PubMed database to investigate RLS in terms of its pathophysiology, genetic associations, and therapeutic options. 

The following keywords were used: restless leg syndrome, iron-deficiency, end-stage renal disease, therapy, treatment, dopamine, and genes​​.

## Review

Primary RLS

Primary, also known as idiopathic RLS, can occur at any age with or without a family history. The exact pathogenesis has not been found, with many researchers hypothesizing a genetic involvement in the disease. A study conducted in 2007 by Winkelmann et al. was the first large-scale genome-wide association study (GWAS) that identified three genomic associations with RLS. The study included MEIS1, BTBD9, and MAP2K5/SKOR1 genes located within these loci and their association with RLS that was subsequently confirmed in several follow up studies [14]. In 2011, Catoire et al. reported that there is an association between MEIS1 and RLS. The findings were based on the research that was performed on Caenorhabditis elegans, a type of roundworm. The results also revealed that the worms with a deficiency of iron had a reduced amount of MEIS1 [15]. In 2012, DeAndrade et al. examined the gene BTBD9 on mutant mice. The study revealed that the BTBD9 was associated with RLS and altered the serum iron levels and monoamine neurotransmitters [16]. In 2018, a GWAS study was conducted on 646 patients with chronic insomnia disorder (CID) with or without RLS. It was one of the first studies on the direct role of the MEIS1 role as a prime cause of RLS. The patients were profiled for the three MEIS1 variants and were grouped as CID without RLS and CID+RLS. The study revealed a strong association of MEIS1 as the prime cause of RLS in all patients [17]. A similar study was conducted on the Chinese population in 2017, targeting different variants of genes. A total of 116 Chinese individuals suffering from RLS were enrolled with 200 control subjects to determine the relationship between genetic factors and primary RLS. The results showed a single nucleotide polymorphism (SNP) variant of MAP2K5/SKOR1 as a prime cause of RLS in the Chinese population [18]. A meta-analysis of the Asian population, in general, revealed that two SNPs of BTBD9 cause a higher risk of RLS [18]. These findings show the genetic nature of RLS and the role of different SNPs in its prevalence [19]. However, how these genes impact different racial groups is still unknown and more data are needed. Some studies indicate that the North American and white populations are more susceptible to RLS as compared to the African and Asian populations [20].

Other variants are found to be associated with RLS as well. Studies conducted in 2008 concluded that there is an association between neuronal nitric oxide synthase (nNOS1/NOS1) genes that generate nitric oxide (NO) and RLS and attention deficit hyperactivity disorder (ADHD) in children [21]. The NO acts directly on the central nervous system and is involved in pain perception and the sleep-wake cycle [22]. This study provides evidence for an association of variants in the NOS1 gene and RLS and suggests the involvement of the NO/arginine pathway in the pathogenesis of RLS [22]. A deficiency or mutation in the NOS1 gene results in RLS [23]. The results were supported by a case-control study of 918 Caucasian patients with RLS [24]. In 2015, Jiménez-Jiménez et al. tested this theory with 205 Spanish patients with RLS to determine if there is an association between RLS and NOS1. However, the results concluded that there was no significant association between NOS1 and RLS [25]. Based on these studies, it may be concluded that the association of NOS1 with RLS may be correlated with specific demographics; however, we need further research to validate it [25].

Secondary RLS

Unlike primary RLS, secondary RLS is independent of family history, age, or racial profile. Secondary RLS occurs in response to some already existing neurological or nutrient deficiency-related diseases. It may also be a symptom of another disease or illness, such as end-stage renal failure, diabetes, pregnancy, undiagnosed ADHD, or iron deficiency [26]. One of the most common etiologies associated with secondary RLS is iron deficiency in the brain tissue. This concept has been supported by several CSF studies that show symptom improvement with iron supplementation [27]. It is unclear if iron directly correlates with RLS as iron is necessary for tyrosine hydroxylase activity, which is the rate-limiting step in dopamine synthesis [28]. A 2009 study reported it as the "iron dopamine model’’ in which the iron deficiency levels in the brain tissues cause abnormalities in the dopaminergic system, causing the RLS [29]. In 2013, Oexle et al. studied 954 individuals suffering from RLS to determine if there was an association between iron-related genes and RLS. The results revealed that the association was weak [30]. García-Martín et al. did a study to determine if there is an association between heme oxygenases (HMOX1 and HMOX2 genes) and RLS. The results showed a weak association between both HMOX1 and HMOX2 with RLS [31].

Based on these studies' findings, there appears to be a weak direct correlation between iron deficiency and RLS. While iron deficiency is often present in patients with RLS, it may be a consequence rather than a cause. There is also a weak association between iron-related genes and RLS, thus supporting the idea that any form of iron deficiency may not be a direct cause for RLS. Still, further research is recommended to understand this correlation between RLS and iron fully.

RLS in pregnancy

Iron deficiency is one of the most common causes associated with RLS; however, numerous other conditions may also be linked to RLS, including pregnancy. The prevalence of RLS in pregnancy ranges between 10% and 34%, which is three times higher than in the general population [32]. It is believed that RLS in pregnant women is associated with several factors such as family history, low serum iron, high estrogen level, hormonal changes, vitamin D deficiency, and calcium metabolism [33].

**Figure 1:**
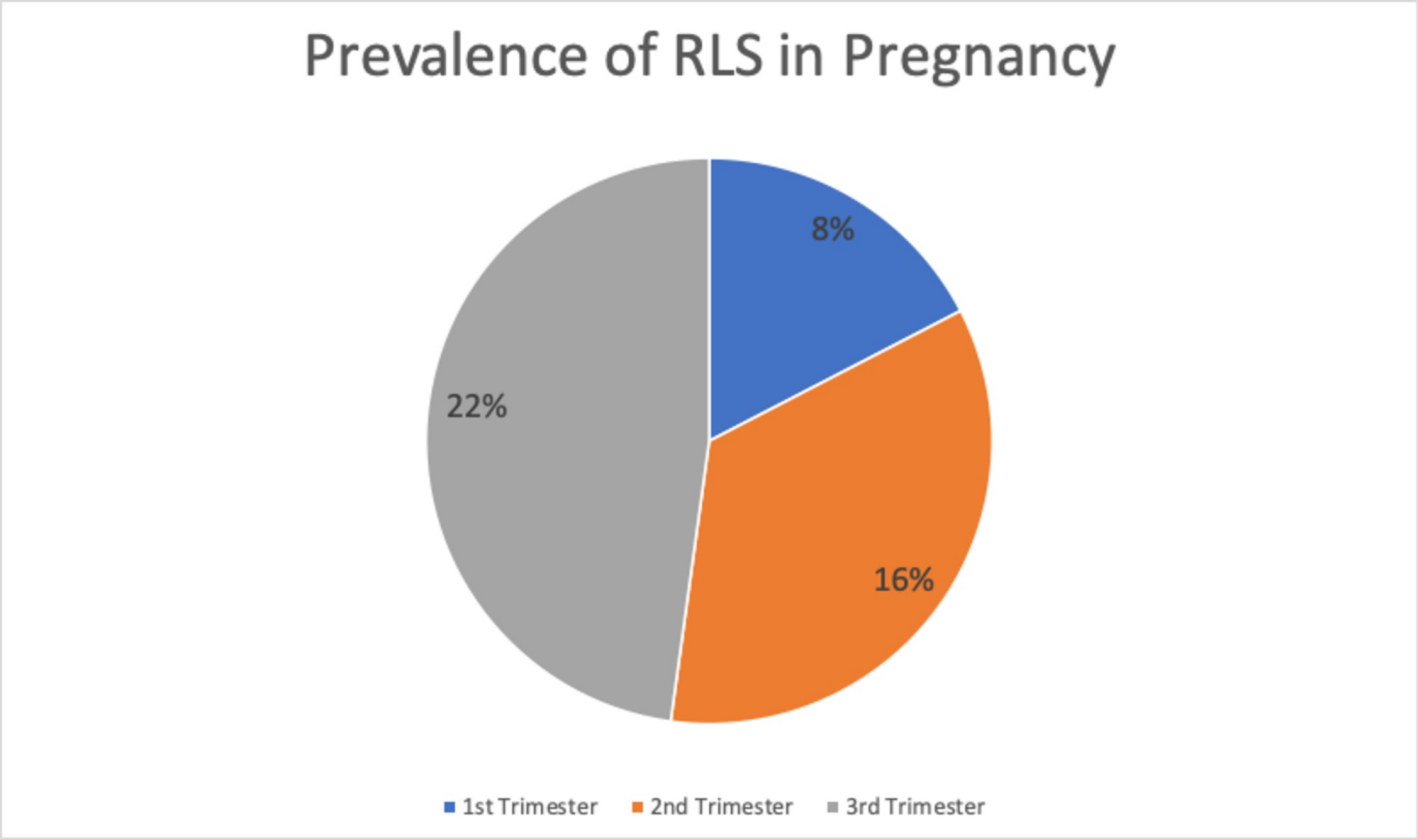
This figure explains the prevalence of restless leg syndrome (RLS) in pregnant women at different stages of the pregnancy. Of all the cases, 22% are reported in women in their third trimester, 16% in the second trimester, and 8% in the first trimester.

Similarly, RLS may also be associated with pregnancy and delivery complications, such as hypertension, cardiovascular disease, and preeclampsia [34]. Symptoms are most frequently present during the third trimester of pregnancy and disappear around delivery [35]. In a study conducted in 2017, researchers reported that the prevalence of RLS was 22% in the third trimester, 16% in the second trimester, and 8% in the first trimester (Figure 1) [36].

**Figure 2:**
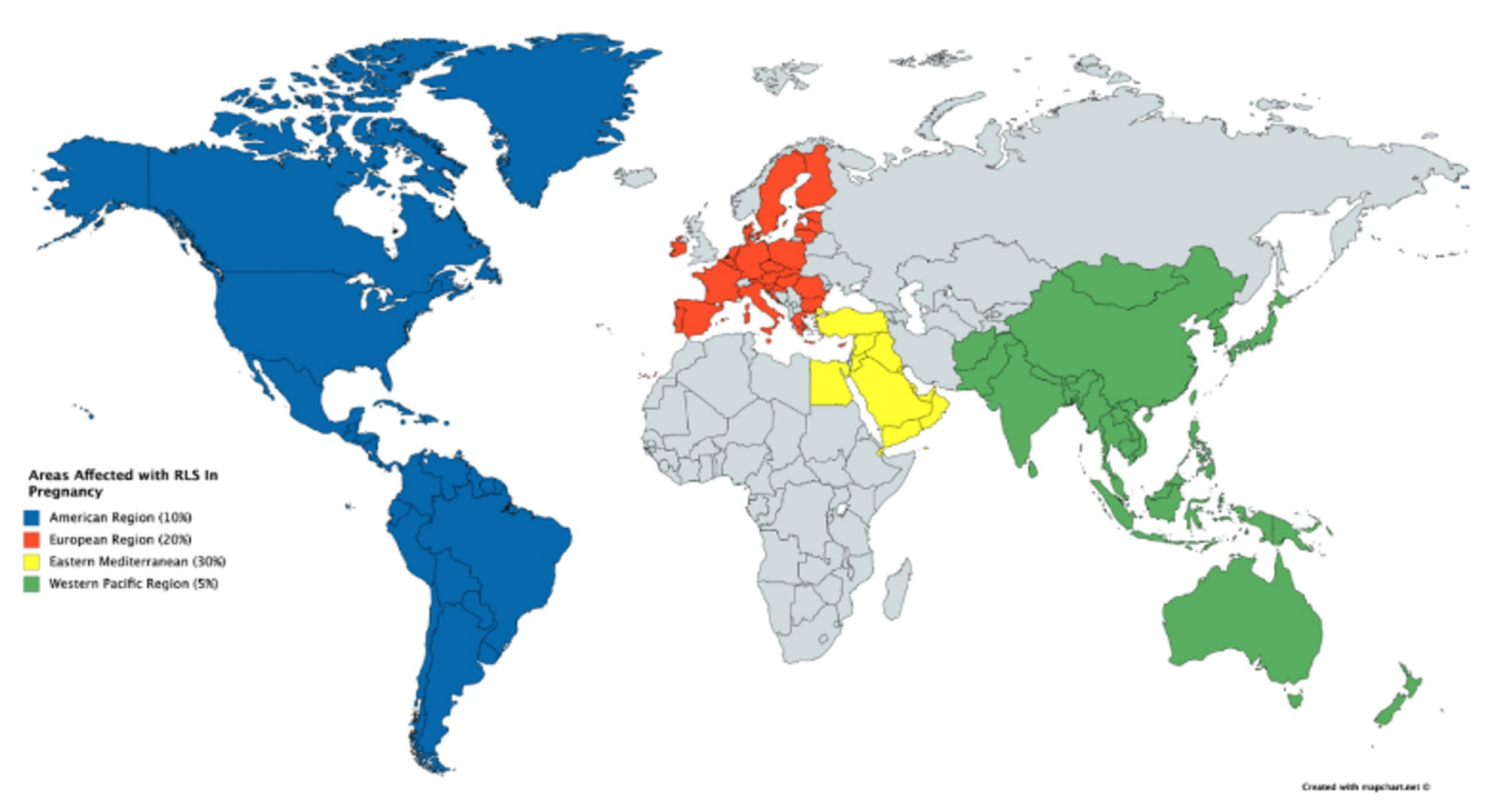
This figure explains the prevalence of RLS in pregnancy in different regions of the world. As already mentioned, RLS is influenced by many factors, heredity and pregnancy-related RLS which is most prevalent in the Eastern Mediterranean region, with 30% of the total cases reported.

The results were supported by a meta-analysis in which 196 articles were analyzed and a total of 51,717 pregnant women were included [36]. Chen et al. also looked at the prevalence of pregnancy-related RLS prevalence in different populations. The prevalence was highest in the Eastern Mediterranean region at 30%, followed by the European region at 22%, the North American region at 20%, and Western Pacific at 14% (Figure 2) [36]. Based on these findings, RLS most commonly appears in the third trimester and East Mediterranean women. The studies were limited by insufficient data for the African and the South-East Asia Region [36]. Because of the limited data on RLS in pregnancy, there are not many medicines that can be used for RLS management in pregnancy [32]. The pathophysiology of RLS in pregnancy remains inconclusive, and further research based on geography is recommended as RLS in pregnancy leads to primary RLS later on.

End-stage renal disease (ESRD)

Another common disease that secondary RLS is associated with is end-stage renal disease (ESRD). The exact pathogenesis is unknown, as there are many possible risk factors associated with it. A multicenter study was conducted on 1130 patients with ESRD in Taiwan to determine the risk factors associated with RLS in ESRD. It concluded that 25.3% of ESRD patients develop RLS. The study also found that RLS with ESRD was even more prevalent among patients presenting with type 2 diabetes, anemia, low serum iron status, and long duration of dialysis [37]. Similar studies conducted on the Saudi population concluded that ESRD patients undergoing dialysis are more susceptible to insomnia, excessive daytime sleep (EDS), and RLS than healthy people of the same age [38]. The study also found that afternoon dialysis was associated with more RLS [38]. In 2018, Ma et al. suggested that the metabolic changes of thalami played an essential role in the onset and progression of secondary RLS and that secondary hyperparathyroidism in patients with ESRD caused RLS [39]. 

Apart from the metabolic changes and possible secondary factors causing RLS in ESRD, genetic variants are also associated. In 2011, Schormair et al. conducted a case-control study of 341 ESRD patients with RLS to determine the association with MEIS1, BTBD9, MAP2K5/SKOR1, and PTPRD. They found that patients who have ESRD have an association with the genetic variant BTBD9 [40]. Jiménez-Jiménez et al. found an association between specific variants of the VDR gene and ADH1B gene with the development of RLS [12,13].

While these are some of the many etiologies of secondary RLS, these studies prove that RLS is not only related to iron deficiency in the brain but also to vitamin D deficiency, alcohol consumption, and other pathological conditions. Further studies are recommended to see which other variants are associated with RLS.

Therapeutic approaches to RLS

Treatment for RLS has always been challenging because of the scarcity of data on the disease pathophysiology. Dopaminergic agonists have always been the first-line agent followed by opioids, anticonvulsants, and iron supplements. The FDA has recently approved dopaminergic agonist variants as a nightly sleep therapy for RLS patients [41]. However, each medication's side effects or shortcomings depend on the individual patient’s type and duration of medication. One of the most challenging issues in the treatment of RLS is the paradoxical worsening of symptoms. The patient will initially respond well to dopaminergic agonists, but the symptoms would subsequently worsen as the same treatment continues [9]. Several studies have been conducted to find an alternative and more efficient way of treating RLS while minimizing adverse effects. As in most cases, iron deficiency was a common factor among all RLS patients, thus supplementing the diet with iron is a safer option for most patients. However, this may have led to adverse effects in pregnant women and patients with ESRD [42]. 

**Figure 3:**
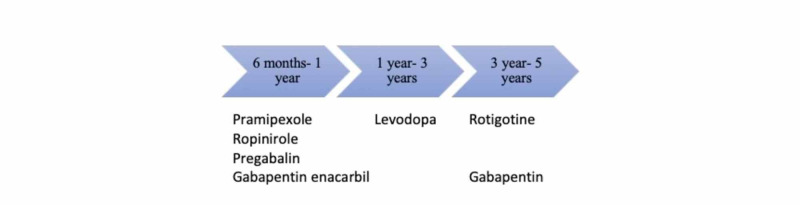
This figure shows the effectiveness of different dopaminergic drug therapies in the treatment of RLS. Usually, the patients go through a period of complete relief, followed by a period of augmentation.

In 2013, Garcia-Borreguero et al. conducted a review to determine the most effective drug for treating RLS. They concluded that gabapentin enacarbil, pramipexole, and ropinirole were effective for up to one year in treating RLS, followed by two years with levodopa and five years with rotigotine (Figure 3) [43]. 

Studies also conclude that the use of dopamine or α-2-δ calcium-channel ligand is recommended as the first-line treatment for RLS along with other agents depending on other factors including, the severity of RLS, cognitive function, and comorbid conditions [43]. In 2018, Faulkner reported that α-2-δ ligands were a better choice for RLS compared to dopamine agonists as α-2-δ ligands were both safer and more effective compared to dopamine agonists [44].

In 2018, Zucconi et al. reported that drugs that work on adenosinergic and glutamatergic neurotransmission seem promising for the treatment of RLS. However, still, further clinical trials and studies are recommended to compare their efficiency to those of first-line agents [45]. Similar studies have established the association between RLS and NOS1 genes and stated that NO modulating agents could provide new treatment options for RLS [25]. 

Other than pharmacological treatment, lifestyle changes are also recommended for patients with RLS. In 2018, a study concluded that RLS symptoms could be significantly reduced through exercise training, transcutaneous spinal direct current stimulation, pneumatic compression devices, light therapy, repetitive transcranial magnetic stimulation, and acupuncture. The authors also reported that exercise training is highly effective in RLS secondary to uremia from ESRD [46]. Endogenous laser ablation would be a better option for patients suffering from RLS with superficial venous insufficiency [46]. In 2019, Harrison et al. conducted a trial of treating RLS using non-pharmacological methods. They found repetitive transcranial magnetic stimulation, exercise compression devices, counterstain manipulation, infrared therapy, and standard acupuncture as effective alternatives to medications, and suggested that these therapies might reduce RLS symptoms [47]. 

Overall, these studies provide us with an alternative and effective way of treating RLS. The non-pharmacological approach may be better than the pharmacological approach, and the α-2-δ ligands may be a better choice than current first-line agents. Still, further research is recommended on adenosinergic, glutamatergic neurotransmission, and NO modulating agents as they may treat RLS more effectively than the current regimens.

Limitations

This article references animal studies and studies only pertaining to certain demographics. Therefore, the conclusions may not be reliable.

## Conclusions

The article is a step toward understanding RLS and its primary causes. Primary genes associated with idiopathic RLS include MEIS1, BTBD9, and MAP2K5/SKOR1. The other genes that are secondary to RLS include alcohol-dehydrogenase 1B (ADH1B) gene, vitamin D3 receptor (VDR) gene, and NOS1 gene. Further research is required as different gene variants may differ from each demographic region. Further research is also required for pharmacologic treatment as some drugs may provide better treatment than its first-line agents. 
